# Toward Community-Based Natural Language Processing (CBNLP): Cocreating With Communities

**DOI:** 10.2196/48498

**Published:** 2023-08-04

**Authors:** Malvika Pillai, Ashley C Griffin, Clair A Kronk, Terika McCall

**Affiliations:** 1 Center for Biomedical Informatics Research Stanford University School of Medicine Stanford, CA United States; 2 Veterans Affairs Palo Alto Health Care System Palo Alto, CA United States; 3 Department of Health Policy Stanford University School of Medicine Stanford, CA United States; 4 Center for Medical Informatics Yale School of Medicine New Haven, CT United States; 5 Division of Health Informatics Department of Biostatistics Yale School of Public Health New Haven, CT United States; 6 Section of Biomedical Informatics and Data Science Yale School of Medicine New Haven, CT United States

**Keywords:** ChatGPT, natural language processing, community-based participatory research, research design, artificial intelligence, participatory, co-design, machine learning, co-creation, community based, lived experience, lived experiences, collaboration, collaborative

## Abstract

Rapid development and adoption of natural language processing (NLP) techniques has led to a multitude of exciting and innovative societal and health care applications. These advancements have also generated concerns around perpetuation of historical injustices and that these tools lack cultural considerations. While traditional health care NLP techniques typically include clinical subject matter experts to extract health information or aid in interpretation, few NLP tools involve community stakeholders with lived experiences. In this perspective paper, we draw upon the field of community-based participatory research, which gathers input from community members for development of public health interventions, to identify and examine ways to equitably involve communities in developing health care NLP tools. To realize the potential of community-based NLP (CBNLP), research and development teams must thoughtfully consider mechanisms and resources needed to effectively collaborate with community members for maximal societal and ethical impact of NLP-based tools.

## Background

### Growth of Natural Language Processing

Natural language processing (NLP) tools are being rapidly developed and diffused at scale. ChatGPT is a chatbot generative pretrained transformer built on a large language model that generates human-like text, which reached over 100 million users in 2 months after launching in November 2022 [1]. Training on enormous quantities of text data, such as websites, articles, or online forums, enables generated conversations to be exceedingly human-like and useful for a variety of tasks (eg, summarization, text generation). However, these tools can also perpetuate stereotypes and misinformation due in part to training data and modeling limitations, as well as human interactions. The pitfalls of NLP tools and their potential negative impact recently led to a call to action from technology leaders and federal governments to pause training powerful artificial intelligence (AI) systems to ensure accurate, transparent, and safe use throughout society [2-4].

### Current State of Health Care NLP

With the emergence of large amounts of health care data, NLP has increasingly been adapted and applied to a range of tasks, including information extraction from clinician notes or social media, patient risk stratification, clinical decision support, and patient-facing chatbots that answer questions. A key component of the NLP pipeline in health care is incorporating stakeholder perspectives in decision-making processes. Recent research has shown the importance of trustworthy ground truth labels for model development. Health care NLP typically relies on subject matter experts (SMEs) who are clinicians or health professionals to incorporate clinical perspectives into biomedical informatics research [5]. These perspectives are usually incorporated during the data annotation phase, where SMEs come to consensus and label data to create a gold standard or benchmark for model validation. A growing body of research on human-in-the-loop NLP suggests integrating feedback during all modeling stages (eg, raw data, annotations, model selection, training, and evaluation) can improve model performance, interpretability, and usability [6]. For example, COVID-19 tweets were analyzed using topic modeling and sentiment analysis, with result interpretation from 2 public health experts. This analysis yielded useful findings on the public’s reaction to the pandemic and feelings around actions to prevent the spread of the virus [7].

### Need for Community Involvement

Commonly, the patient’s voice is not included in the conversation. When a researcher is presented with text written by patients (eg, a message from a patient in the patient portal, posts on social media) or notes written by clinicians (eg, patient lifestyle descriptions), patients can serve as experts by experience (EBEs) in partnership with clinical SMEs. Inclusion of individuals with lived experience from the beginning of the research and development pipeline would be valuable, as they often have insightful ideas and hypotheses and may provide more context on the topic and considerations for development and implementation [8]. For example, the Department of Veterans Affairs (VA) has established several veteran engagement panels where veterans with lived experience act as patient advisors and provide continuous feedback to researchers [9]. For NLP, engaging individuals early in the process would be useful to elicit feedback on model development, explanation, and discussion of model building and tuning. Early community engagement is also beneficial to inform evaluation and recruitment strategies across different communities. As researchers reflect on the implications of their work, individuals who could be impacted by the work should also be engaged for ethical and moral considerations.

Community empowerment, where patients gain more control over decisions regarding their health, is also growing, largely due to information liquidity and access to communication tools arising from the internet and social media [10]. Patients are increasingly interested in how their data are being used and contributing to societal advancements through “citizen science.” However, historical injustices, existing structural barriers, and discriminatory policies have prevented minoritized communities from accessing and understanding health information, having adequate representation in research studies, and being included in the development process of the very tools that are supposedly created to improve health outcomes.

Historically, the field of public health has involved input from the public to address concerns around environmental and social factors that impact the health of communities [11]. For example, in the 1940s two physicians from South Africa moved to a rural region to collaborate and train the local residents as health workers [12]. Their approach centered around community involvement to enhance public health and care delivery strategies. Since this pioneering work, involving communities in research has grown to have a positive influence on health care delivery. For public health interventions, community-based participatory research (CBPR) focuses on actively involving community stakeholders throughout the research process to improve awareness of community needs and cultural expectations to enhance the sustainability of evidence-based interventions [13]. The CBPR framework emphasizes the need to incorporate perspectives from communities who experience health inequities and those who may express distrust in the health care system [14]. Community members can be engaged throughout 5 different stages of the research process (inform, consult, involve, collaborate, and empower). Although this type of community involvement has existed for decades in the public health sciences, it has not yet caught on within the field of NLP.

Including EBEs (eg, patients, community members) in conversations alongside SMEs (eg, health professionals) can increase the translatability of NLP-related works. For instance, an oncologist may know everything there is to know about cancer, but unless they have had cancer themselves, the subjective experience can only be described in relation to what cancer patients have communicated to them. This phenomenon was first outlined by Frank Jackson in his 1982 article “Epiphenomenal Qualia,” describing a scientist existing in a colorless world who has read everything there is to know about color but has not ever seen it [15]. The central question is, Does the scientist gain knowledge when she leaves the colorless world and experiences color for the first time? Patients have been included as SMEs in NLP research but not in a comprehensive way throughout the NLP pipeline, as CBPR proposes. Patients have largely been included as consultants in the past, when they should ideally be integrated into research teams as EBEs alongside SMEs and researchers.

In this perspective, we draw upon the field of CBPR to identify possible ways to involve individuals in the NLP pipeline to facilitate colearning and transparency between community members and research and development teams. Advocacy for including community members in research teams has increased, but the rapid advancements in technology must be paired with amplified patient voices to validate their experiences and ensure equitable tools that represent all patients.

## Toward Community-Based NLP

The full value of applying a participatory research framework to NLP has yet to be realized. “Participatory research prioritizes coconstructing research through partnerships between researchers and stakeholders, community members, or others with insider knowledge and lived expertise” [16]. The traditional approach to NLP includes the processes of selecting raw data, annotating the data, selecting the model, training the model, then evaluating and deploying the model. This process is often completed by health informaticians, health care professionals, data scientists, developers, engineers, and other individuals with computational skills, excluding participation from the communities that the end product intends to benefit. The results are models that are ineffective in delivering the desired results due to lack of context and cultural considerations. True CBPR (1) acknowledges the community as a unit of identity, (2) builds on the community’s strengths and resources, (3) promotes colearning and cocreation with the community, (4) seeks to balance research and action so that the collaboration is mutually beneficial in advancing the research agenda and the community’s needs, (5) highlights the importance of community-defined problems, (6) establishes an iterative process to develop and maintain partnerships between the researchers and community, (7) disseminates knowledge gained from the project to all stakeholders (eg, community members, researchers), and (8) plans for a long-term commitment to the work and the community that it will serve [17].

Community members should be involved as much as possible in NLP system development.

**Figure 1:**
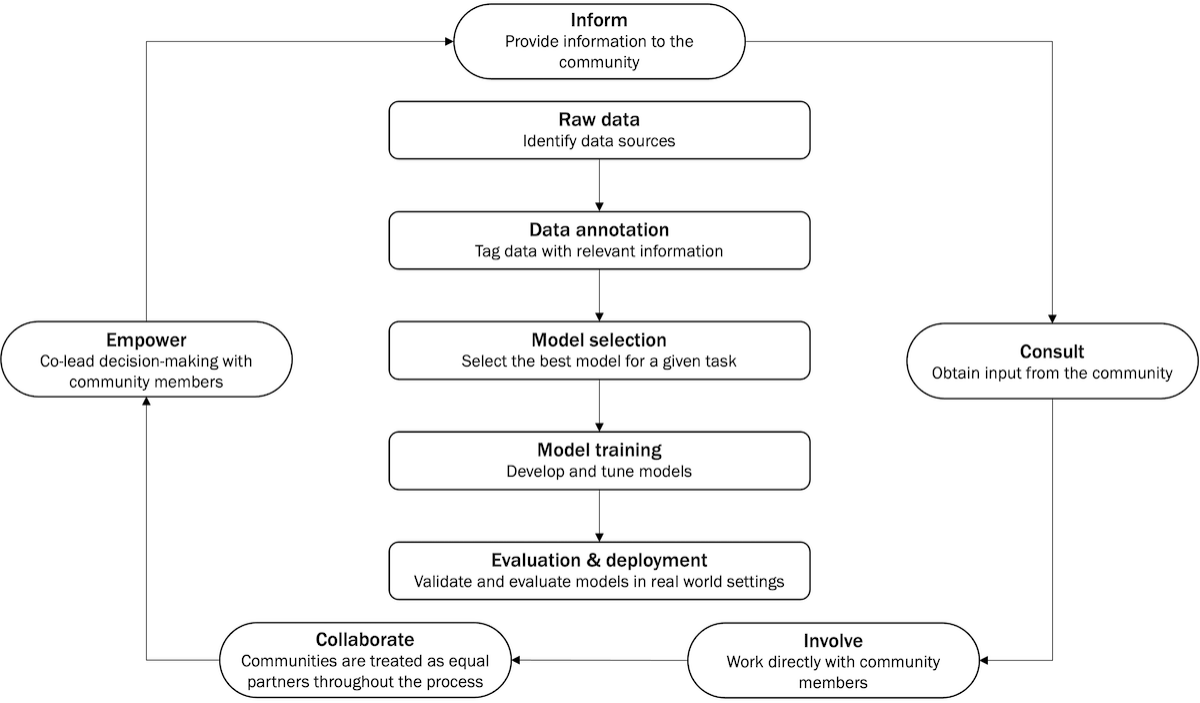
Community-based natural language processing framework.

There will likely be high variability in the level of involvement, depending on factors such as one’s desires and background in research and development, time commitment, compensation, and project-specific factors. Based on prior work by Kwon and colleagues [14] that focused on the application of core principles of CBPR in the development of patient-centered outcomes in research, we seek to propose recommendations on how to apply CBPR practices to the NLP pipeline. The community-based natural language processing (CBNLP) framework consists of 5 principles based on the Public Participation Spectrum [14,18]: (1) inform—provide information to the community; (2) consult—obtain input from the community (eg, interviews, focus groups); (3) involve—ensure researchers work with the community throughout the research process (eg, community advisory boards); (4) collaborate—consider communities as partners in research (eg, train individuals to be coresearchers); and (5) empower—promote community co-led decision-making, which can be integrated into each stage of the NLP pipeline (Figure 1, Table 1).

**Table 1:** Recommendations for engaging community members in a community-based participatory research approach to natural language processing.

	Raw data	Data annotation	Model selection	Model training and testing	Deployment and validation
Inform	Provide information about potential data sources; describe the data source origination and curation	Give a description of the annotation process and how it is used for natural language processing development	Provide an overview of models being considered in the project	Create tutorials and educational resources	Describe translating natural language processing models into real-world settings, with implications on the potential risks, benefits, and impacts
Consult	Meet with community members to elicit feedback on data source selection; discuss any questions or concerns related to the data source(s)	Gather diverse views and thoughts on the annotation guidelines	Ask community members about their perspectives on the models being considered	Obtain feedback on the goals of the model (eg, interpretability)	Gather input on perceived feasibility, utility, outcomes, and deployment strategies
Involve	Identify meaningful data sources; discuss assumptions or concerns of each source	Include community members in the development and refinement of annotation guidelines	Discuss models and alternatives	Engage community members in the model training process to ensure the model is training as intended	Include community members in discussing considerations for equity and potential failures
Collaborate	Consider community members as partners when selecting data sources through ongoing and open discussions	Work together throughout the annotation process	Partner with community members during model selection and weigh model tradeoffs together	Jointly work with community members during model training with continuous discussions of goals and progress	Work together during the predeployment testing, refinement, and deployment phases with ongoing discussions around safety and efficacy
Empower	Provide the opportunity for community members to vote on data source decisions	Promote shared decision-making	Support community members in voting to select models best suited to the task	Engage community members in setting priorities for model training and testing	Allow community members to set goals and make decisions around model deployment and validation

### Inform

#### Opportunities

From the beginning, community members should be involved in defining the most important issue(s) that will be the focus of the research collaboration. Consensus should be gained before proceeding with NLP activities. The inform principle focuses on providing information to community members about the types of data that may be used, how the data will be used, how the data will be stored and protected, and potential benefits and harms in using the data for developing the system. For example, information about potential data sources to be used in the project (raw data), including their origin, curation, and any potential biases or unfairness that can result from their use, should be transparently shared with community members [19,20]. Additionally, community members should be given a description of the annotation process and how it is used for NLP development. During the model selection process, community members should be provided with an overview of models being considered in the project, including the strengths and weaknesses of each model. Furthermore, community members should be given an overview of the process for translating NLP models into real-world settings, with implications related to the potential risks, benefits, and impacts during deployment and validation.

#### Challenges

Despite the growing interest in NLP, it is important to acknowledge that community members may not have the same background and training in NLP methodologies as research and development teams. As a result, there will be a learning curve involved in comprehending NLP-related information, such as NLP model underpinnings. Tutorials and educational resources in lay terms should be created iteratively with community members to facilitate learning about the NLP processes at basic literacy levels. For example, NLP can be explained in terms of tools that patients may be more familiar with, such as editing suggestions in Microsoft Word or autocomplete in emails.

### Consult

#### Opportunities

The consult principle focuses on obtaining input from community members (eg, interviews, focus groups). Acknowledging the community as a unit of identity, individuals with lived experience should be consulted to obtain diverse perspectives and facilitate more robust research by unveiling previously unseen angles. The research and development team should meet with individuals to elicit feedback on data source selection and discuss any questions or concerns related to the data source(s). Data source discussions can be facilitated through “data set nutrition labels,” which provide a description of the data content, quality, and representation [21]. For example, when trying to detect depression from patient-generated texts like emails, community members may have concerns about the racial and ethnic diversity of the group of patients who wrote the emails, since depression can be experienced and expressed differently by people of different cultures. Additionally, consulting with community members with lived experience can provide diverse views and thoughts on the annotation guidelines by allowing for conversations about interpretation of the data and context. Recent studies have illustrated the importance of high-quality labels, as label quality directly impacts the reliability of a tool [22]. Colearning and cocreation with the community should be prioritized, and community members should be asked about their perspectives on the models being considered during the model selection phase as well. The research and development team should also seek feedback on the goals of the model (eg, interpretability, accuracy, sensitivity, specificity), and gather input on perceived feasibility, utility, outcomes, and deployment strategies. Without feedback from the community, it is difficult to understand how useful a model would be in practice.

#### Challenges

In addition to NLP-specific resources, community members would need to be familiar with health care data sources (eg, social media, electronic medical records) and contexts to understand potential pitfalls. Accessible infographics and pamphlets summarizing background information should be created to facilitate understanding. There must also be an emphasis on consulting with a diverse group of community members; it has historically been an issue that purposive recruiting is not performed for biomedical and public health research studies.

### Involve

#### Opportunities

The involve principle focuses on researchers working with the community throughout the research process (eg, community advisory boards). Building on the community’s strengths and resources, the research and development team should work with community members to identify meaningful data sources and discuss assumptions or concerns for each source. Community members with lived experience should also be included in the development and refinement of annotation guidelines and discussion of models and alternatives to gather input on ethical and accurate models. Furthermore, community members should be engaged in the model training process to ensure the model is training as intended. Community members should also be included in developing a plan for validation and deployment, including consideration for equity and potential failures. “What-if” tools, which visualize model behavior across different data subsets and scenarios, can be used to help research and development teams and community members better understand model outputs and fairness [23]. For more advanced models of engagement in NLP development, individuals may desire more advanced roles, including being involved in the decision-making process. For example, in the data annotation phase, for an investigator analyzing social media data for sentiment analysis, the text can be written in different vernaculars (eg, Black Twitter, Muslim Twitter, *Subtle Curry Traits*). Individuals who understand the meaning of phrases and the emotion reflected in the text could be involved in developing and refining data annotation guidelines.

#### Challenges

To involve community members in each phase of the NLP pipeline, iterative processes to develop and maintain partnerships between the researchers and community should be established. Maintaining academic-community partnerships requires that community members be equitably compensated for their time and effort. Compensation has historically been an issue (eg, undercompensating participants from minoritized communities), which prevents equitable participation in research and development studies. Prior to launching a project, researchers should have transparent discussions with community members on defining roles, responsibilities, and addressing any questions or challenges. Scheduled follow-up conversations should also be conducted to check in with community members throughout the project lifecycle.

### Collaborate

#### Opportunities

The collaborate principle focuses on considering communities as partners in research (eg, training individuals to be coresearchers). The research and development team should seek to balance research and action so that the collaboration is mutually beneficial in advancing the research agenda and the community’s needs. Community members with lived experience should be considered as partners when selecting data sources and included in ongoing and open discussions. Working and cocreating together throughout the annotation process, including developing the guidelines and discussion of annotation disagreements, improves the accuracy and applicability of results. For example, community members could conduct open coding to identify major concepts within specific corpora, and these concepts could be used to inform annotation guidelines. Community members can also serve as arbitrators in case of disagreements on annotations to foster deeper discussions on the meaning of the data based on their backgrounds and experiences. Partnering with community members during model selection and weighing model trade-offs together increases transparency and builds trust. Patients can help set priorities that necessitate the use of specific techniques (eg, interpretable vs black box algorithms). Moreover, the research and development team should collaborate with community members during model training and continuously discuss goals and progress. In model training and evaluation, the patient perspective can be critical in analyzing model predictions and providing perspectives on how the system can be safely deployed for patients or care teams. Based on the use case, patients would be able to articulate their preferences for how performance should be optimized. For example, researchers may find that sensitivity is more important for a research task, while patients may find that specificity is a more important metric. There should also be ongoing discussions around safety and efficacy during the predeployment testing, refinement, and deployment phases.

#### Challenges

Tools that can facilitate successful academic-community collaboration should be used or produced. For example, when considering an annotation task, interfaces should be user-friendly, adhere to accessibility standards, and be amenable to cultural tailoring (eg, support various languages or custom vocabularies). Documentation should also be produced throughout collaborations to ensure that findings have a lasting impact beyond individual projects and permeate subsequent research endeavors. The documentation can also present evidence of the impact of community engagement in NLP research. It is difficult to quantify the benefits and pitfalls of community engagement; however, prior research in NLP crowdsourcing described potential psychological harms of annotations on crowdworkers [24]. In biomedical research, community members may encounter troubling content, and they should be made aware of the risks and potential harms ahead of time. While they are members of the research team, they must also be protected, just like research participants.

### Empower

#### Opportunities

The empower principle includes the most involvement from community members and focuses on prioritizing community members as coleaders in the decision-making. Community members with lived experience should be invited to vote on data source decisions. Additionally, the research and development team should promote shared decision-making during the annotation process and support community members in selecting models best suited to the task. This process requires taking the time to educate community members on important factors to consider and providing resources in lay terms to enhance understanding of processes. Empowering the community to share in decision-making requires capacity building within the community to participate in setting priorities and decisions for model training, testing, deployment, and validation. Additionally, community members should be empowered to lead the dissemination of knowledge gained from the project. For example, they should not only inform decisions on what venues and formats should be used to share the insights from the project but also be encouraged to lead these activities with the support of the research and development team. Community members should also feel empowered to lead in the decisions to establish plans for a long-term commitment to the work and the community that it will serve. For example, there could be a timeline for updates on project progress, and after project completion, results and any continuing efforts should be shared with community members. There must be consensus on this plan for continuity and mechanisms that facilitate accountability to the commitment to the community.

#### Challenges

Empowering community members to become coleaders involves creating an environment where they feel comfortable and valued and fostering their active participation in the decision-making process. Members should feel at ease expressing dissenting viewpoints if necessary. There may be power dynamics between clinical SMEs and EBEs that could impact community members’ comfort levels. Mitigation strategies and safeguards to protect community members should be considered. Researchers may also face an additional challenge in that community members should not feel exploited, which entails demonstrating dedication to sustainable research that will give back to communities.

## Benefits and Considerations for Community-Based NLP

### Benefits

Community member and patient participation in biomedical research as EBEs has strong potential to improve research quality and health outcomes. A PubMed search for publications with the term *community-based participatory research* within the last 5 years returned over 2500 results. The use of CBPR methods has become more ubiquitous as the research community has been charged to think more about how their work promotes health equity and addresses health disparities. Most studies that used CBPR methods to improve participation of racial and ethnic minoritized communities in clinical trials had positive outcomes [25]. Moreover, community participation in an advisory committee and input on data collection, intervention development, and recruitment efforts for studies were associated with positive outcomes [25]. Community participation also has greater implications for patient engagement in health care, as it can open up dialogues that provide more opportunities for patient-centered care. With growing mistrust in systems collecting big data, patients have become more interested in how their data are being used. Being transparent with and working with patients on data-driven research can help build trust and demonstrate how critical their data are for advancing community well-being and health care delivery. Through training and education, patients could become more actively engaged in their own health and better understand how researchers use patient data to improve their quality of care. As NLP models are increasingly being developed and implemented in health settings, collaboration among patients, caregivers, care teams, and researchers is valuable to help broaden perspectives and facilitate discussion of preferences, values, and needs across these stakeholder groups. As such, this supports mutual learning and knowledge sharing across these groups. Collaboration between patients with similar experiences may also foster stronger community connections and social support.

### Ethical Considerations

Community members participating in NLP research must be protected just like those participating in other health care research (eg, clinical trials). Institutional review board approval should be obtained prior to conducting any research projects involving participants. The ethical principles of the Belmont Report—respect for persons, beneficence, and justice—should not only guide the implementation of biomedical research projects but also govern the entire process. Community advisory board members should sign a confidentiality agreement and disclose conflicts of interest. They should also receive honorariums for their efforts.

### Resource and Training Considerations

Realizing the value of CBNLP requires new ways of thinking about how individuals from the community are involved in model development and research. This approach necessitates a shift in the current attitudes of researchers toward traditional research participants, as researchers must consider participants as partners in research rather than as subjects or interviewees within their cohort. New procedures and protocols must be created to effectively integrate individuals and their input throughout the pipeline. Capacity building and equipping community members with the necessary training, skills, and resources to meaningfully contribute are vital. Incorporating human-computer interaction principles and methods into the development of CBNLP interfaces should be considered, including accessibility, user experience, and usability. A survey of NLP-related crowdsourcing tasks on Amazon Mechanical Turk found that technical and instruction issues have a big role in influencing the quality of data from the workers [26].

Funding mechanisms should also be considered to compensate individuals for their time and contributions. Having participants coauthor papers, present at conferences, and give talks in the community can also be a way to honor their contributions. However, special emphasis should be placed on limiting burdens on community members. Beyond compensation, community members should feel empowered to voice their comfortableness with being involved in all activities, and their decisions must be respected. Additionally, involving community members throughout NLP activities requires new strategies for developing efficient research pipelines that do not prolong the time to discovery or implementation of tools into care or communities.

### Equity Considerations

Individuals engaged in research advisory groups (eg, community advisory boards) may not be representative of the larger community, so it is important to ensure recruitment of a diverse group of community members. Careful attention should be made to avoid discouraging participation from individuals who may be less interested in research or technology. A CBNLP approach may not be appropriate for participation of all community members or practical for all types of projects, such as those involving sensitive or triggering topics.

### Technical Considerations

With respect to algorithm development and use, there are considerations for incorporating stakeholder perspectives and weighing input during decision-making [19,27]. Once a representative group of EBEs are established, it also becomes necessary to weigh their expertise against the expertise of SMEs. This weighting is especially crucial due to propagation and proliferation of health care misinformation and disinformation. Despite the prevalence of misinformation among members of the general public, SMEs often have some gaps in knowledge or have entrenched implicit biases. For example, gaslighting of women patients by health care providers is still prevalent, and many providers endorse pseudoscientific beliefs about Black patients [28-31]. Recently, misinformation and disinformation ontology and vocabulary research has become more prevalent in informatics. Many machine learning–based methodologies for detection of false information have been tested, but these systems do not yet appear to be used in evaluation by SMEs or EBEs.

An ideal scenario would be to have a population of hybrid SME-EBEs, such as oncologists who themselves have had cancer. Having such individuals involved would help establish a weighting mechanism between SME information and EBE information. However, in practice, there may not be many individuals that have both the subject matter expertise and lived experience. An alternative approach would be to construct data sets that weigh experience and expertise in a systematic manner, which can then be used to generate a model for qualification as an SME, an EBE, a hybrid SME-EBE, or as a nonexpert.

This weighting algorithm would have to be subject- and population-specific and would require extensive surveying. For example, jury learning has been proposed to integrate different voices into machine learning models where data labelers are considered jurors, and the jury learning architecture repeatedly samples a jury to produce a median outcome over multiple trials [32]. This weighting procedure can be critical when considering opposing expert opinions. Over time, several other factors may also impact research, such as language and knowledge change, which can be mitigated by data resources. However, data resources like diachronic corpora (ie, corpora with text from different periods of time) or ontologies need to be of appropriate size and representativeness, and they must be able to be augmented in the future through a reproducible process. Assuming this is the case, these changes can help make fusion and integration processes more reproducible.

## Conclusions

Continuing to create digital tools without adequate representation of communities that will be affected by their use to identify risks and make decisions on resources can result in the automation of discrimination [33]. In the era of rapid development and AI adoption, developing partnerships between research and development teams and community members is essential for equitable technology advancement. The advent of ChatGPT in the mainstream has increased the number of people familiar with these technologies, which may have a positive impact on involving communities in NLP research. It also highlights the need to understand how people are using these emerging technologies and their perspectives on their use in health care [34]. In turn, this can lead to improved tools, experiences, and health outcomes for all members of society. Drawing upon CBPR and traditional health care NLP processes, we identify and examine possible ways to equitably involve communities. Some recommendations could be implemented more readily, such as providing individuals with information about how their data are used in an NLP project. Other recommendations will take more time and resources, including collaborating with individuals to develop an annotation schema and annotate corpora.

The paradigm shift in health care NLP will require thoughtful consideration to recruit, train, and empower community members to make meaningful contributions. Careful considerations should be made to avoid overburdening individuals by ensuring comfortableness, honoring their perspectives and opinions, and providing fair and continuous compensation. Harmonizing the contributions from EBEs with those of SMEs must also be weighed to produce maximum beneficial impact on quality of care. By cocreating with communities, for communities, we can foster inclusion, innovation, and discovery through community-based NLP.

## Abbreviations

CBNLP: community-based natural language processing
CBPR: community-based participatory research
EBE: expert by experience
NLP: natural language processing
SME: subject matter expert
